# Wavelet Screening: a novel approach to analyzing GWAS data

**DOI:** 10.1186/s12859-021-04356-5

**Published:** 2021-10-07

**Authors:** William R. P. Denault, Håkon K. Gjessing, Julius Juodakis, Bo Jacobsson, Astanand Jugessur

**Affiliations:** 1 Department of Genetics and Bioinformatics, Norwegian Institute of Public Health, Oslo, Norway; 2 Centre for Fertility and Health, Norwegian Institute of Public Health, Oslo, Norway; 3 Department of Global Public Health and Primary Care, University of Bergen, Bergen, Norway; 4 Department of Obstetrics and Gynecology, Institute of Clinical Sciences, Sahlgrenska Academy, University of Gothenburg, Gothenburg, Sweden

**Keywords:** SNP, GWAS, Multiple testing, Polygenic association, Wavelet regression

## Abstract

**Background:**

Traditional methods for single-variant genome-wide association study (GWAS) incur a substantial multiple-testing burden because of the need to test for associations with a vast number of single-nucleotide polymorphisms (SNPs) simultaneously. Further, by ignoring more complex joint effects of nearby SNPs within a given region, these methods fail to consider the genomic context of an association with the outcome.

**Results:**

To address these shortcomings, we present a more powerful method for GWAS, coined ‘Wavelet Screening’ (WS), that greatly reduces the number of tests to be performed. This is achieved through the use of a sliding-window approach based on wavelets to sequentially screen the entire genome for associations. Wavelets are oscillatory functions that are useful for analyzing the local frequency and time behavior of signals. The signals can then be divided into different scale components and analyzed separately. In the current setting, we consider a sequence of SNPs as a genetic signal, and for each screened region, we transform the genetic signal into the wavelet space. The null and alternative hypotheses are modeled using the posterior distribution of the wavelet coefficients. WS is enhanced by using additional information from the regression coefficients and by taking advantage of the pyramidal structure of wavelets. When faced with more complex genetic signals than single-SNP associations, we show via simulations that WS provides a substantial gain in power compared to both the traditional GWAS modeling and another popular regional association test called SNP-set (Sequence) Kernel Association Test (SKAT). To demonstrate feasibility, we applied WS to a large Norwegian cohort (N=8006) with genotypes and information available on gestational duration.

**Conclusions:**

WS is a powerful and versatile approach to analyzing whole-genome data and lends itself easily to investigating various omics data types. Given its broader focus on the genomic context of an association, WS may provide additional insight into trait etiology by revealing genes and loci that might have been missed by previous efforts.

## Background

The main objective of a genetic association study is to identify the genes and loci that are associated with a phenotype of interest. Although the human genome is very similar across individuals, it is interspersed with single base-pair differences called single-nucleotide polymorphisms (SNPs) that collectively account for the observed differences across individuals. One of the most common approaches to genetic association testing is to conduct a genome-wide association study (GWAS), where the significance of the effect of each SNP on a phenotype is assessed in a sequential fashion. Despite its many successes, this approach has two important limitations: (i) it incurs a substantial multiple-testing burden due to the large number of tests carried out simultaneously, and (ii) it ignores the genomic context of an association by failing to exploit the typically dense microarray-based genotyping of the genome. As larger regions of the genome are more likely to contribute to the phenotype [1], considering the effect of one SNP at a time would not efficiently model how a larger change in the genome might impact the phenotype.

The issue of multiple testing can be resolved using a regularization method such as Fused Lasso [2], which allows a penalized regression to be performed. It can also take into account how variables (here SNPs) located near each other might produce similar effects. Fused Lasso can thus be used to define a region of association between a group of SNPs and the phenotype. However, the main disadvantage of Fused Lasso is that it can only perform local testing, whereas it may be more judicious to test for associations over larger regions of the genome.

Despite an increased interest in penalized regressions within the broader statistical community, they remain elusive in the top-tiered genetic publications. Penalized regression has recently been incorporated into PLINK [3], one of the leading software for GWAS, but the lack of a comparable software for meta-analysis is a major drawback of this approach. A comprehensive genome-wide association meta-analysis (GWAMA) typically involves the analysis of summary statistics from multiple cohorts, and although such meta-analyses are now feasible in the Lasso regression setting [4], they are not currently available for variants of Lasso regression or other regularization penalties.

Several regional tests have been developed for GWAS, including the Burden test [5, 6], C-alpha [7], and SKAT [8]. These tests were primarily designed for the analysis of rare variants. However, a few of the more recent developments, including SKAT-O [8], can handle both rare and common variants. As these tests are not specifically designed to pinpoint the exact location of the region harboring the association, they have to be applied to relatively small regions. As a result, the total number of tests needed to perform a genome-wide screening still remains too large and intractable.

To address these shortcomings, we developed a new approach called Wavelet Screening (WS) for analyzing genome-wide genotype data by leveraging key insights from functional modeling [9, 10]. Specifically, we adapt the approach of Shim and Stephens [10] to test for association with a functional phenotype (the response signal) by first transforming the signal using Fast Discrete Wavelet Transform (FDWT) [11] and then testing for single-SNP associations. In essence, we reverse the approach of Shim and Stephens [10] by modeling SNP signals as wavelets over large regions of the genome followed by a regression of the wavelet coefficients on the phenotype.

The use of reverse regression to search for genetic associations is becoming more widespread in the genetic literature (see [12] for an example). Our approach treats sizable chunks of the genome ($\approx 1$ million base pairs) as the functional phenotype and provides a broader test by enabling an estimation of the fraction of wavelet coefficients (blocks) associated at each level of depth. A dimensional reduction is then performed using wavelet transform before testing for association between the wavelet coefficients and the phenotype. This broader approach to testing combined with multiple levels of information may provide additional insight into the reason for detecting a genetic association. Furthermore, by reversing the regression and targeting a region for association testing, we use regional association instead of single-SNP association to reduce the number of tests to be performed. Specifically, using overlapping windows of 1 Mb in length reduces the number of tests from eight million (for common SNPs) to approximately 5000. We propose screening regions of 1 Mb in size to cover most of the linkage disequilibrium (LD) blocks that are present in a given population, irrespective of ethnicity [13].

The remainder of this paper is structured as follows. We first describe the statistical setting of the different analyses, including the wavelet methodology used to generate the wavelet coefficients. Next, we describe our test statistic between the wavelet spectrum and the phenotype $\Phi$. In the current context, $\Phi$ represents a univariate vector of either a continuous, countable, or binary trait. After a comprehensive evaluation of WS by a series of simulations, we showcase its application using a large dataset from the Norwegian HARVEST study—a sub-project nested within the Norwegian Mother, Father and Child Cohort Study (MoBa) [14]. Our primary phenotype of interest is gestational duration.

## Materials and methods

### Haar wavelet transform

**Fig. 1:**
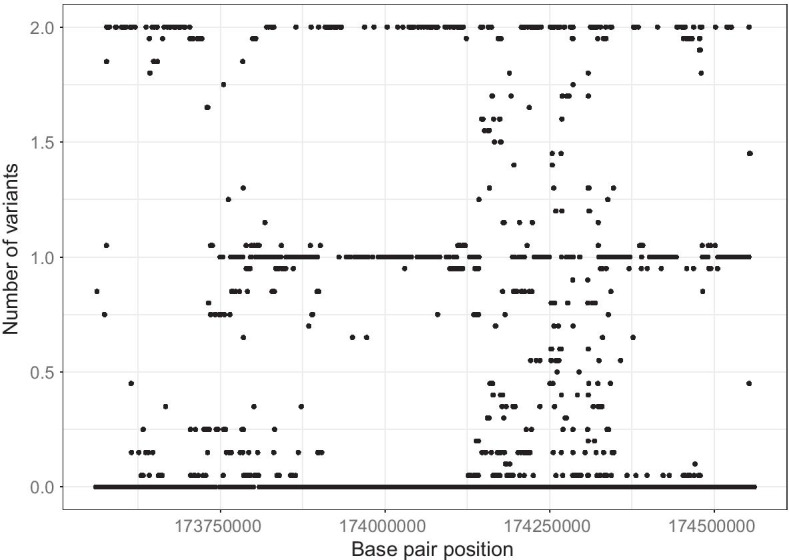
Genetic variation in one individual within a locus spanning two million base pairs (2 Mb), including 10,000 imputed SNPs

Our method transforms the raw genotype data similarly to the widely used ‘Gene- or Region-Based Aggregation Tests of Multiple Variants’ method [15] (Fig. 1). Like the Burden test, the effects of the genetic variants in a given region are summed up to construct a genetic score for the regression. The first step in our analysis is the application of FDWT to the multi-SNP data. In the next subsection, we introduce the Haar wavelet transform and show how the wavelet coefficients are computed. Readers unfamiliar with wavelets are referred to a comprehensive introduction by Nason [16]. In the rest of this article, ‘wavelet’ specifically refers to the Haar wavelet.

We code a SNP 0 if an individual is homozygous for the reference allele (usually assigned to the more frequent or ‘major’ allele), 1 if heterozygous, and 2 if homozygous for the alternative allele (the less frequent or ‘minor’ allele). This is the standard way of coding alleles in an additive genetic model [3]. Let $G_{0,k}(bp)$ denote the ‘true’ genetic signal of individual *k* at physical position *bp* (base pair), and let $G_{k}(bp)$ be the observed, imputed version of $G_{0,k}(bp)$. We assume that

1 $$\begin{aligned} G_{k}(bp)&= G_{0,k}(bp)+ \epsilon _{k}(bp) \end{aligned}$$

where $\epsilon _{k}(bp)$ are independently and identically distributed (*iid*) over individuals, with $\mathrm {Var}(\epsilon _{k}(bp))=\sigma ^2(bp)$. The variance $\sigma ^2(bp)$ at position *bp* can be interpreted as a function of the imputation quality *IQ*(*bp*), which has a value in $\left[ 0,1\right]$. 1 represents a perfectly imputed SNP or genotyped SNP; hence, $\sigma ^2(bp) \propto 1-IQ(bp)$. As the data used here were already quality-controlled, only SNPs with an $IQ\in \left[ 0.8,1\right]$ were retained for further analyses. We assume that the imputation metrics are independent and heteroscedastic over *bp*. As the value of a SNP is in $\{0,1,2\}$ and then in $\left[ 0,2\right]$ according to the dosage convention after imputation [3], the distribution of $\epsilon _{k}(bp)$ is not straightforward. However, as our model is calibrated by simulations, this error distribution does not have to be specified.

We define a genetic region $GR_{lb,ub}$ (GR, genetic region; lb, lower bound; up, upper bound) on a given chromosome as the set of physical positions *bp* in the interval $lb< bp < ub$. In the rest of the paper, we assume the analyses are performed within a fixed genetic region $GR_{lb,ub}$ on a given chromosome. We observe the value of $G_{k}(bp)$ at pre-determined and increasing positions $bp_1,..., bp_n$ within the interval (*lb*, *ub*), with some error due to the genome-wide imputation process [17]. For now, we assume having $n = 2^J$ equally spaced observations within $GR_{lb,ub}$ and denote the observed value of $G_{k}(bp_i)$ by $g_k(bp_i)$; i.e., the data value measured on individual *k* at position $bp_i$, $i = 1, \ldots n$, where the $bp_i's$ are equally spaced. We define wavelet *d* and *c* coefficients as sequences of length $2^J$. These coefficients are computed by Mallat’s pyramid algorithm [11].

For the coefficients at the highest scale (i.e., scale $J-1$), for $i \in \{1,\ldots ,2^{J-1}\}$,

$$\begin{aligned}&d_{J-1,i}= g_{k}(bp_{2i}) -g_{k}(bp_{2i-1})\\&c_{J-1,i}= g_{k}(bp_{2i}) +g_{k}(bp_{2i-1}). \end{aligned}$$

These coefficients correspond to local differences (or sums) of the measured values. For lower scales, the coefficients are computed as follows:

$$\begin{aligned}&d_{j-2,i}= c_{j-1,2i} -c_{j-1,2i-1}\\&c_{j-2,i}= c_{j-1,2i} +c_{j-1,2i-1}. \end{aligned}$$

Finally, the coefficients at the lowest scale (i.e., scale 0) are computed as:

$$\begin{aligned} d_{0,1}=c_{0,1}= \sum _{i=1}^{2^J} g_{k}(i). \end{aligned}$$

These procedures are often written as square matrices $W_d$ and $W_c$ (d and c procedures, respectively) of size $2^J$, where the rows of $W_d$ and $W_c$ are normalized. We have $d = Wg_{k}$ and $c = W' g_{k}$. In addition, because the matrix $W_d$ is orthogonal, we have:

$$\begin{aligned} ||d ||^2= \left( Wg_{k}\right) ^t Wg_{k} =||g_{k} ||^2. \end{aligned}$$

Using the $2^J$ wavelet coefficients for individual *k*, all values $g_{k}(bp)$ in the genetic region $GR_{lb,ub}$ can be completely recovered. However, this wavelet transformation assumes that the data are evenly spaced and that there are $n = 2^J$ measurements, which may not be realistic in practice. To avoid this assumption, we use the method of Kovac and Silverman [18], which is briefly explained in the “Pre-processing of data” section.

#### Wavelet representation of the genome

In essence, the coefficients obtained after performing wavelet transform on a genomic region can be viewed as local ‘scores’ of the genotype, with the following interpretations:

- At scale 0, the wavelet coefficients *d* and *c* can be interpreted in the same way: they summarize the discrepancy between an individual’s genotypes and the reference genotypes coded as 0...0. This is essentially the test comparison performed in standard gene or regional tests.
- The wavelet $d_{s,l}$ coefficient at scale $s > 0$ and location *l* for an individual represents the difference in the number of minor alleles between the left part of the region (defined by *s*, *l*) and the right part.
- The wavelet $c_{s,l}$ coefficient at scale $s > 0$ and location *l* for an individual represent**s** the discrepancy between an individual’s genotypes and the reference genotypes coded as $0\ldots 0$ for the region defined by *s*, *l*.

The main rationale behind this modeling is that, if a genetic locus has an effect on the phenotype, then the association is likely to be spread across genomic regions of a given size (scale) at different positions (locations). By using wavelet transform to perform a position/size (or, alternatively, time/frequency) decomposition and then regressing the wavelet coefficients on the phenotype, one can visualize *where* (location) and *how* (scale) the genetic signal influences the phenotype.

In the rest of this article, we use ‘wavelet coefficients’ to refer to *c* coefficients specifically. *c* coefficients are easier to interpret than *d* coefficients. For instance, in case of completely observed genotypes, *c* coefficients correspond to the sum of minor alleles, similar to the Burden test [19].

### Pre-processing of data

#### Non-decimated wavelet transform

We use the method of Kovac and Silverman [18] to handle non-decimated and unevenly spaced data. This method takes an irregular grid of data, for example, the sampling of different genetic regions, and interpolates the missing data into a pre-specified regular grid of length $2^J$. For a given genetic region $GR_{lb,ub}$ with measurements at *n* positions $bp_1 ... bp_n$, we map this region into a (0, 1) interval using the affine transformation $x\xrightarrow {} \frac{x-bp_1}{bp_n}$. We then define a new grid of points of length $2^J$ on (0, 1) as: $t_{0},\ldots ,t_{N-1}$, where $N=2^J, J\in {\mathbb {N}}$, $t_{k}= (k+\frac{1}{2})2^{-J}$ and $J = min\{j \in {\mathbb {Z}}, 2^j \ge n \}$. We interpolate the mapped signal into this grid and run wavelet transform to obtain the wavelet coefficients. In practice (see the “Applications” section), we recommend selecting genetic regions with a relatively high density of imputed SNPs.

#### Coefficient-dependent thresholding and quantile transform

For each individual wavelet decomposition, we use the VisuShrink approach [18] to shrink the interpolated wavelet coefficients and reduce the dependence between the wavelet coefficients within scales. This allows an estimation of the variance of each wavelet coefficient before determining a specific threshold for each wavelet coefficient. We can account for the individual heteroscedasticity of the noise by determining specific coefficient-dependent thresholds using the variance of the wavelet coefficient. Next, we quantile-transform the distribution of each wavelet coefficient within the population to make sure that each distribution follows a *N*(0, 1) distribution. As we use the quantile-transformed wavelet coefficient as the endogenous variable (see “Modeling” section), the above transformation ensures that, under the null hypothesis, the residuals are normally distributed. This also controls for spurious associations resulting from any deviation from the Normal distribution assumption of a linear model.

### Modeling

In essence, our approach to modeling aims at detecting regions containing sub-regions associated with a trait/phenotype of interest. We localize these sub-regions to ease the interpretation of the output. We first need to assess whether certain scales are associated with the phenotype at different locations to estimate the effect of a genetic region on the phenotype of interest. Within a genetic region, let ${\tilde{G}}_{sl}$ denote the quantile-transformed wavelet coefficient at scale *s* and location *l*. To test for association between the phenotype and the wavelet coefficient, we regress the wavelet coefficients on the phenotype $\Phi$ using the traditional least squares estimation for Gaussian linear models [20]. To adjust for covariates *C* that may be confounders in the GWAS, we incorporate the covariates into the regression models. The regression models for each scale and location are defined as follows:

2 $$\begin{aligned}&M_1: {\tilde{G}}_{sl} = \beta _{sl,0} + \beta _{sl,1}\Phi +\beta _{sl,C}C+\epsilon \end{aligned}$$

where *C* is a matrix of dimension $c \times 1$ and $\beta _{sl,C}$ is a matrix of dimension $1 \times c$, and $\epsilon \sim N\left( 0,1\right)$. We compute the association parameters $\beta _{sl,1}$ of the wavelet regression for each pair (*s*, *l*) using least squares estimation [20].

#### Evidence towards the alternative

For a given locus, a genetic signal might be assumed to occur in only a subset of the regression coefficients. Thus, the ${\hat{\beta }}_{sl,1}$ may be viewed as originating from a mixture of two Gaussian distributions, each representing a specific hypothesis:

- Under $H_0$ the ${\hat{\beta }}_{sl,1}$ are distributed as $N(0,\sigma ^2_{null})$.
- Under $H_1$ some ${\hat{\beta }}_{sl,1}$ are distributed as $N(\mu _{alt},\sigma ^2_{alt})$.

To help identify a subset of $\beta$ that convey the signal, we fit a mixture of the two Gaussian distributions to the collection of estimated ${\hat{\beta }}_{sl,1}$, assuming mixture weights $1 - \pi _{alt}$ and $\pi _{alt}$, respectively. Under the null hypothesis, the full mixture is not identifiable. To estimate $\sigma ^2_{null}$, $\mu _{alt}$, and $\sigma ^2_{alt}$ in all cases, and to ensure that the estimated ${\hat{\pi }}_{alt}$ becomes small under $H_0$, we constrain the Gaussian mixture fitting using a modified version of the EM algorithm with the restriction that ${\hat{\sigma }}^2_{null} > \frac{\left( X^tX \right) ^{-1}_{2,2}}{10}$ and ${\hat{\sigma }}^2_{alt}>k\left( X^tX \right) ^{-1}_{2,2}$, where *X* is the design matrix with the phenotype $\Phi$ in the second column and *k* is of the order of 100.

After obtaining the estimates, we compute—for each beta coefficient—the posterior probability ${\hat{\pi }}_{l,s}$ of $H_1$ knowing $\beta _{s,l}$ by

3 $$\begin{aligned} {\hat{\pi }}_{l,s} ={\mathbb {P}} \left( H_1|{\hat{\beta }}_{sl,1} \right)&= \frac{{\hat{\pi }}_{alt,s,l}\phi \left( {\hat{\beta }}_{sl,1};{\hat{\mu }}_{alt},{\hat{\sigma }}^2_{alt} \right) }{{\hat{\pi }}_{alt,s,l}\phi \left( {\hat{\beta }}_{sl,1};{\hat{\mu }}_{alt},{\hat{\sigma }}^2_{alt} \right) +\left( 1-{\hat{\pi }}_{alt,s,l}\right) \phi \left( {\hat{\beta }}_{sl,1};0,{\hat{\sigma }}^2_{null} \right) }, \end{aligned}$$

where $\phi \left( \cdot ;\mu ,\sigma ^2\right)$ is the density of a Gaussian distribution, with mean $\mu$ and variance $\sigma ^2$. To reduce the influence of betas that most likely belong to $H_0$, we propose a thresholding of the posterior probabilities ${\hat{\pi }}_{l,s}$ that decrease with sample size as well as wavelet depth. Based on the work by Donoho and Johnstone [21], we define the thresholded probabilities by

4 $$\begin{aligned} {\tilde{\pi }}_{l,s}= \max \left( {\hat{\pi }}_{l,s} - \frac{1}{\sqrt{2log(n)}\sqrt{2^s}},0\right) . \end{aligned}$$

We later use the ${\tilde{\pi }}_{l,s}$ to localize the sub-regions of interest (for details, see the “Model and results” section).

Finally, we compute the average evidence towards the alternative by

5 $$\begin{aligned} L_h = \sum _{s=0}^{S} \frac{1}{2^s} \sum _{l=1}^{2^s} {\tilde{\pi }}_{alt,s,l}\phi \left( {\hat{\beta }}_{sl,1};{\hat{\mu }}_{alt},{\hat{\sigma }}^2 _{alt} \right) -\left( 1-{\tilde{\pi }}_{alt,s,l}\right) \phi \left( {\hat{\beta }}_{sl,1};0,{\hat{\sigma }}^2_{null} \right) . \end{aligned}$$

**Fig. 2:**
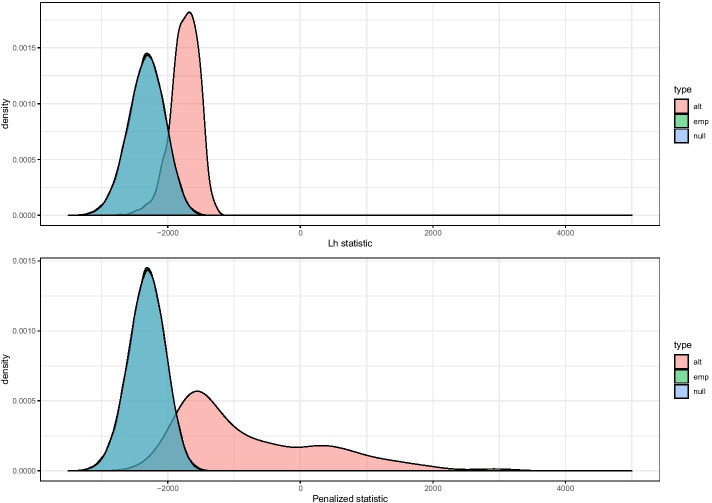
Average evidence towards the alternative hypothesis. The simulated null distribution is shown in blue (’null’), the empirical null distribution in green (’emp’), and the alternative distribution in pink (’alt’)

Note that, in contrast to the work of Shim and Stephens [10], where lower scales have more weight, our test statistic applies equal weight to each scale. As shown in the upper panel of Fig. 2, the separation between the null and alternative distributions achieved by this test statistic ($L_h$) alone is not optimal because the resulting test power is low. For additional details, see the “Complex genetic signal” section and the “Appendix”.

#### Inflation of the alternative hypothesis

To improve the separation of the null and alternative distributions, we extract two additional pieces of information from the ${\tilde{\pi }}_{l,s}$. First, we compute the average proportion of associations per scale. The proposed test statistic is a weighted sum applying the same weight to each scale. This can be interpreted as an alternative horizontal summary of the association:

6 $$\begin{aligned} p_h = \sum _{s=0}^{S} \frac{1}{2^s} \sum _{l=1}^{2^s} {\tilde{\pi }}_{alt,s,l}. \end{aligned}$$

Second, we extract a vertical summary by considering sub-regions of the overall screened region. We divide the region of association into $S-1$ sub-regions, where *S* is the maximum depth of analysis. We summarize the proportion of associations vertically, and for each average, we consider the positions that overlap with the sub-regions. For example, the first coefficient at scale 1 contributes half of the sub-region average of association.

7 $$\begin{aligned} p_v&= \sum _{k=1}^{S-1} \frac{1}{n_k} \sum _{s=1}^{S} \sum _{l= \big \lfloor \frac{2^S (k-1)}{S-1} \big \rfloor }^{\big \lfloor \frac{2^S k}{S-1}\big \rfloor } {\tilde{\pi }}_{alt,s,l} \end{aligned}$$

8 $$\begin{aligned} n_k&=Card\left( (s,l),\forall s \in \left[ 1,S\right] , l \in \llbracket \Bigg \lfloor \frac{2^S (k-1)}{S-1} \Bigg \rfloor ,\Bigg \lfloor \frac{2^S k}{S-1}\Bigg \rfloor \rrbracket \right) \end{aligned}$$

We use the new summaries of association to increase the separation between the null and the alternative by assuming that, under the alternative hypothesis, $p_v$ and $p_h$ tend to be larger. We then build our full test statistic $T_{S_\lambda }$, which requires calibration of the hyperparameter $\lambda$:

9 $$\begin{aligned} T_{S_\lambda }&= L_h + \lambda \cdot min(p_h,p_v) \end{aligned}$$

A larger $\lambda$ would yield higher power if we assume that $p_v$ and $p_h$ tend to be larger under the alternative hypothesis. However, increasing $\lambda$ can also change the shape of the null distribution. Assuming that the null distribution is normal, we use this as a fitting criterion to select the hyperparameter.

#### Calibration of the hyperparameter and statistical significance

**Fig. 3:**
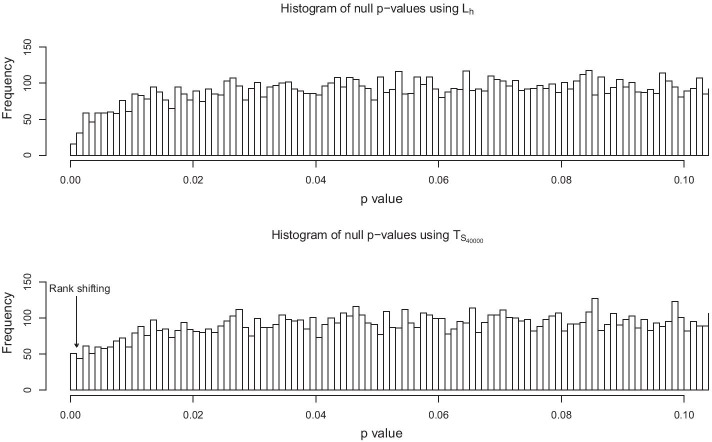
Rank shifting of the bin of interest

Our goal is to find the right balance between having as large of a $\lambda$ value as possible while keeping the null distribution normal. As $min(p_h,p_v)$ is not normally distributed (bounded distribution), the larger $\lambda$ is, the further $T_{S_{\lambda }}$ deviates from normality. To strike the right balance, we simulate $L_h$ and $min(p_h,p_v)$ under the null. Once simulated, we compute $L_h$ and $min(p_h,p_v)$ for each simulation ($10^{5}$ simulations in our case). Next, we fit a normal distribution on $L_h$ and use this fit to generate the histogram of the p-values of the simulations for 1000 bins. We compute the number of counts in each bin and rank them by count (Figs. 2 and 3). We are particularly interested in the rank of the first bin, as an inflation of this bin would influence the false discovery rate. This procedure is repeated for increasing values of $\lambda$, and the search is stopped when a rank increase in the first bin is observed. We select the largest $\lambda$ that results in the rank of the first bin to be equal to the rank of the first bin for $L_h$, denoted as $\lambda ^*$. Finally, we use the normal fitting of $T_{S_{\lambda ^*}}$ to perform the testing. In the “Appendix”, we provide a pseudo code description of the procedure (see Algorithm 1).

As wavelet transform induces a correlation between the ${\hat{\beta }}_{sl,1}$, it is not possible to simulate them from a univariate normal distribution using their theoretical null distribution. One option is to compute the empirical covariance matrix of the ${\hat{\beta }}_{sl,1}$ and simulate $\beta _{sl,1}$ using this empirical null distribution. A second option is to simulate wavelet coefficients using random signals from a normal Gaussian distribution and then re-scale them to obtain a mean of zero and variance of one. A third possibility is to compute the covariance matrix of these wavelet coefficients and re-scale them using the theoretical null distribution of the ${\hat{\beta }}_{sl,1}$. Similar results are achieved using these different options (see Fig. 1 in the “Appendix” for a comparison). In the “Appendix”, we also provide a pseudo code description of the procedure (see Algorithm 2 and Algorithm $2^{ bis}$).

## Results

### Complex genetic signal

We performed simulations catering to a complex genetic signal by combining real genetic data with a simulated phenotype. We used a previously identified locus for gestational age in the Norwegian HARVEST dataset [14] and maternal genotype data from a region on chromosome 7 spanning nucleotide positions 40504539–41504539 based on the human genome build GRCH37-hg19 [22]. This region contains a total of 5209 SNPs in our dataset. An example of the genetic variation in a single individual is displayed in Fig. 1. We performed two sets of simulations to mirror local polygenic effects. Each set of simulation considered different local polygenic effects by SNP location, as follows:

- *High LD Simulations*: we computed the correlation structure (LD) of the above-described locus and identified 28 small LD-blocks. In this simulation set-up, all the SNPs used in constructing the phenotype are selected within high LD regions. These simulations are engineered to mimic a diluted effect of SNPs within different LD blocks, also known as “block polygenic effect”, where each variant has a small additive effect.
- *Random LD Simulations*: all the SNPs (from 1 to 28) used in constructing the phenotype are taken uniformly at random from within the considered locus. These simulations are engineered to mimic a diluted effect of SNPs regardless of the LD structure, where each variant has a small additive effect.

**Fig. 4:**
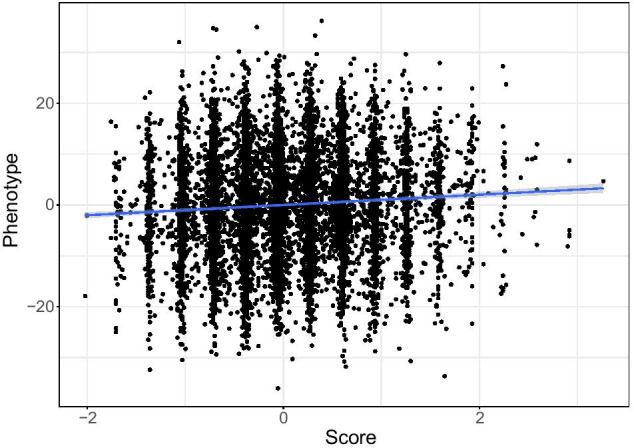
Simulated phenotype against the generated score (20 SNPs selected)

We provide an example of such a phenotype in Fig. 4. In addition to the above simulations, we considered the following two models, each of which mimics different underlying locus effects:

- *Mono-directional model (MD)*: for each iteration, we randomly selected 1 to 28 SNPs, and for each individual, we summed up their SNP dosages within the selected set of SNPs to construct a score. On top of the individual scores, we added normally-distributed noise, scaled so that the genetic score explains $0.5\%$ of the total phenotypic variance.
- *Random direction model (RD)*: the same setting as above, but the sign of the effect (positive/negative) for each SNP is random. In the mono-directional simulations, any additional variant may increase the level of the phenotype. This is not necessarily the case with the random direction model. Taken together, these simulations showcase the sensitivity of WS to the direction of the SNP coding.

The variance explained by a single SNP varies between $0.5\%$, which is typical for the top SNPs in a GWAS [1]), to $0.018\%$, a level at which variants are not normally detected by the standard GWAS framework. We performed simulations for different sample sizes (1000, 5000, 10,000), and for each set-up, we performed 10,000 simulations and ran WS. In addition, we performed $2\times 10^{6}$ simulations for the scenario of no association to assess the type 1 error for each sample size (see Table 3).

Further, we performed $10^6$ simulations of $L_h$ and $min(p_h,p_v)$ for each simulation set-up and searched for the hyperparameter for each sample size (see “Calibration of the hyperparameter and statistical significance” section). As displayed in Fig. 2, there is a good match between the simulation and permutation distributions.

We also compared our method with one of the most popular regional methods for association testing called SNP-set (Sequence) Kernel Association Test (SKAT) [19]. For each sample size, type of effect, and number of SNPs, we performed 1000 simulations. SKAT aggregates individual SNP effects into a regional score before testing for association. We applied SKAT to the same region of 1 Mb as with WS to allow a comparison of the simulation results between SKAT and WS. SKAT is generally recommended for regions between 5 to 25 kb [19]. To run SKAT on a larger region, we used fast-SKAT [23] which allows an efficient computation of p-values.

In Tables 1 and 2, *NA* stands for ‘not applicable’. As the standard single-SNP GWAS does not take LD structure and local polygenicity into account, the only effect modeled here is the dilution effect. As we obtained similar results, we chose not to display them in Tables 1 and 2, but only specified the column *Model* with *NA* for SKAT. Overall, the results in Tables 1 and 2 show that WS is an attractive alternative to single-variant modeling in traditional GWAS. The dilution effect in both tables appeared to be highly non-linear for the GWAS linear modeling, with a steep elbow-shaped curve. In contrast, the power of WS decreases roughly linearly with the number of components in the score for *Random LD Simulation* but increases in the case of *High LD Simulation*. The power of SKAT increases with increasing number of causal SNPs. Compared to WS, SKAT is more powerful for large sample sizes and when a large number of variants affect the phenotype. Conversely, WS has higher power especially for smaller sample sizes and when a moderate number of SNPs affect the phenotype. For $n=1000$, however, none of the methods were optimal. As seen in Tables 1 and 2, WS has non-zero power compared to both SKAT and the GWAS based on single-variant modeling.

**Table 1:** Power of the different methods depending on the number of components in the ‘Random LD Simulations’ (dilution effect)

Size	Model	Method	Significance	1	2	3	4	5	6–10	11–15	16–20	> 20
1000	MD	WS	$1\times 10^{-5}$	0.4	0.5	0.8	1.0	1	0.7	0.6	0.7	0.7
1000	RD	WS	$1\times 10^{-5}$	0.8	0.1	0.0	0.3	0.2	0.5	0.9	0.2	0.4
1000	NA	SKAT	$1\times 10^{-5}$	0.0	0.0	0.0	0.0	0.0	0.1	0.2	0.8	1.8
1000	NA	GWAS	$5\times 10^{-8}$	0.0	0.0	0.0	0.0	0.0	0.0	0.0	0.0	0.0
5000	MD	WS	$1\times 10^{-5}$	9.2	11.1	9.1	6.1	4.6	3.5	2.5	2.3	2.3
5000	RD	WS	$1\times 10^{-5}$	9.6	9.3	7.3	5.4	5.1	2.6	1.4	1.7	1.6
5000	NA	SKAT	$1\times 10^{-5}$	0.0	0.2	0.57	0.8	2.8	7.14	26.2	54.5	82.0
5000	NA	GWAS	$5\times 10^{-8}$	45.5	25.8	16	3.1	2.7	2.8	1.1	0.6	0.3
10,000	MD	WS	$1\times 10^{-5}$	48.1	46.5	44.4	45.1	44.1	31.0	24.6	21.2	21.2
10,000	RD	WS	$1\times 10^{-5}$	49.1	45.2	46.3	47.2	39.2	27.2	15.6	17.2	15.9
10,000	NA	SKAT	$1\times 10^{-5}$	0.2	1.1	1.3	4.4	7.3	32.0	71.6	92.8	99.2
10,000	NA	GWAS	$5\times 10^{-8}$	100	81.8	75.0	68.8	30.6	28.1	15.5	9.6	3.3

For *Random LD Simulation*, Table 1 shows that the GWAS based on single-variant modeling is superior when up to five SNPs are considered and none of the SNPs are located within different LD blocks or are in LD with each other. For a sample size of $n=5000$, WS outperformed the other two methods when at least four SNPs are considered in the simulation. For $n=10{,}000$, the GWAS based on single-variant modeling proved to be the best method when only 1-4 SNPs are considered. As is evident from Table 2, WS has higher power in case of five SNPs or more within different LD blocks.

**Table 2:** Power of the different methods depending on the number of components in the ‘High LD Simulations’ (dilution effect)

Size	Model	Method	Significance	1	2	3	4	5	6–10	11–15	16–20	> 20
1000	MD	WS	$1\times 10^{-5}$	0.9	1.1	0.7	0.9	0.6	0.8	0.6	0.9	0.9
1000	RD	WS	$1\times 10^{-5}$	1.2	0.9	0.2	0.7	0.8	0.5	0.6	0.4	0.8
1000	NA	SKAT	$1\times 10^{-5}$	0.0	0.0	0.0	0.0	0.0	0.1	1.4	4.6	19.8
1000	NA	GWAS	$5\times 10^{-8}$	0.0	0.0	0.0	0.0	0.0	0.0	0.0	0.0	0.0
5000	MD	WS	$1\times 10^{-5}$	17.3	18.3	19.1	20.6	24.0	20.9	19.4	19.3	20.2
5000	RD	WS	$1\times 10^{-5}$	7.3	10.5	8.8	7.1	9.4	8.5	9.2	8.0	9.3
5000	NA	SKAT	$1\times 10^{-5}$	0.0	0.2	1.4	3.0	5.9	26.7	69.5	95.4	99.9
5000	NA	GWAS	$5\times 10^{-8}$	45.5	25.8	16	3.1	2.7	2.8	1.1	0.6	0.4
10,000	MD	WS	$1\times 10^{-5}$	54.8	70.9.	86.8	90.1	92.2	95.4	93.6	94.1	96.5
10,000	RD	WS	$1\times 10^{-5}$	57.3	49.8	54.6	53.8	56.1	54.4	50.7	50.6	52.1
1000	NA	SKAT	$1\times 10^{-5}$	0.0	1.1	6.5	15.6	24.9	64.5	96.5	100.0	100.00
10,000	NA	GWAS	$5\times 10^{-8}$	100	81.8	75.0	68.8	30.6	28.1	15.5	9.6	3.2

MD, mono-directional model; RD, random directional model; NA, not applicable

The increase in power for a single SNP, as shown in Tables 1 and 2, can be explained by the fact that the considered SNP for *High LD Simulation* is within an LD block, which is not necessarily the case with the *Random LD Simulation*. As it is rare that only a single SNP is responsible for the association observed with the phenotype, in a general setting WS would be more powerful than the standard GWAS based on single-variant modeling. More importantly, our simulations show that WS has higher power for detecting an association in case of moderate *‘local polygenicity’*. Overall, our simulations show that the type I error is well-controlled by WS for the proposed simulation settings (see Table 3).

### Applications

Besides the above simulations, we also tested the applicability of WS by performing: 1) a chromosome-wide association study of human gestational duration using real data from a Norwegian cohort [24], and 2) a genome-wide association study of maize grain yield using a dataset from a European plant consortium [25].

### Gestational age

Gestational duration is a complicated phenotype to study because of large measurement errors ($\approx 7$ days [26]) and typically small genetic effects ($\approx 1.5$ days [24, 27]). We used genome-wide genotypic data from mothers in the Norwegian HARVEST study [14] to see if we could replicate the lead SNPs reported by Zhang and colleagues [24] in the hitherto largest GWAMA on gestational duration. These lead SNPs are located on chromosome 5, near a gene called Early B cell factor 1 (*EBF1*). Using the same exclusion criteria for SNPs and individuals as in Zhang *et al. * [24], the lowest p-value obtained in our dataset was $2.8\times 10^{-6}$ for $n=8006$, which is not statistically significant in the traditional GWAS setting.

#### Definition of regions and choice of resolution for gestational age

Although a typical GWAS can now interrogate millions of SNPs at a time, several chromosomal regions are still difficult to analyze due to poor marker density, particularly regions located near telomeres and centromeres, regions containing highly repetitive DNA, and regions of low imputation quality. Most SNPs with low imputation quality are routinely discarded during quality control. Given that we pre-processed the gestational duration data using an interpolation, we tried to avoid analyzing purely interpolated regions by including an additional criterion in the pre-processing step to exclude these types of regions. We propose studying regions of size 1 Mb, with a maximum distance of 10 kb between any two adjacent SNPs. Furthermore, we defined overlapping regions to avoid having a signal located at the very boundary of two regions. By applying these additional criteria, we excluded $18\%$ of the SNPs and defined 248 regions on chromosome 5.

Besides avoiding fully-interpolated regions, we also need to choose an appropriate depth of analysis for the wavelet decomposition. The precision of the wavelet coefficient depends on the number of non-interpolated points in a given region [18]. As a rule of thumb, we propose aiming for 10 SNPs on average for each wavelet coefficient. Following this criterion, the median spacing between any given pair of SNPs was 202 bp in our dataset. This means that, if we divide each locus of 1 Mb into $2^9 =512$ sub-regions, we would, on average, have $\frac{10^6}{2^9}\times \frac{1}{202} \approx 9.7$ SNPs per sub-region. Moreover, this prevents us from having a small number of possible values for the wavelet coefficients of the highest resolution. Having a small number of possible values for the wavelet coefficients would imply that the wavelet coefficients would behave as a discrete variable, and, therefore, the proposed linear model would no longer be appropriate.

#### Model and results

**Fig. 5:**
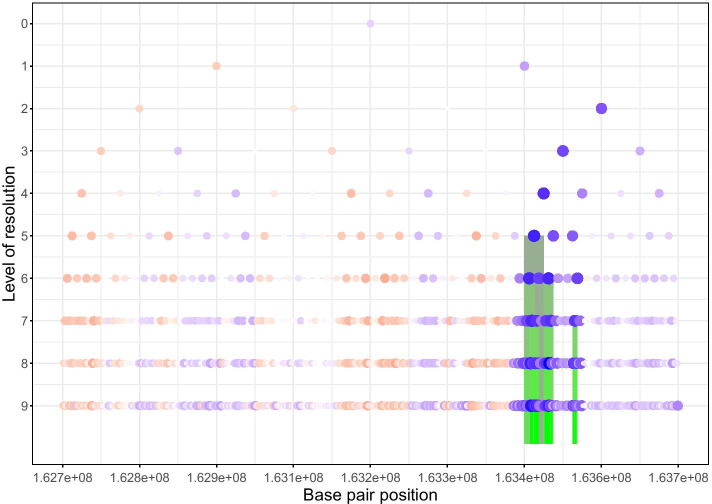
Locus discovered by Wavelet Screening. The dots of different sizes represent the absolute values of the estimated ${\hat{\beta }}_{1,sl}$; blue for negative, red for positive. The highlighted vertical bars represent ${\hat{\pi }}_{sl}$ non-thresholded to zero

We applied WS to the gestational duration dataset described above. We simulated $L_h$ and $min(p_h,p_v)$ $10^5$ times under the null using an empirical correlation matrix. Using these simulations and the steps described in the “Calibration of the hyperparameter and statistical significance” section, we obtained $\lambda ^* =696552$. We then fitted a normal distribution on $L_h +\lambda ^*min(p_h,p_v) +\lambda ^*min(p_h,p_v)$. This distribution was then used to compute the p-values for each locus. These analyses identified two significantly associated loci, as shown in Fig. 5, but because we employed half-overlapping windows, these loci were in fact identical (their respective p-values are $1.1\times 10^{-24}$ and $8.7\times 10^{-7}$). Reassuringly, our results showed that the main SNP in the published GWAMA by Zhang and co-workers is located less than 1 Mb from the locus near *EBF1* detected by WS.

**Table 3:** Estimated Type I error for different sample sizes

	$\alpha$ level					
N	0.0500	0.0100	0.0010	0.0001	0.00001	0.00001
1000	0.050470	0.009669	0.000852	0.000017	0.000006	0.000003
5000	0.049319	0.008993	0.000730	0.000085	0.000013	0.000003
10, 000	0.050709	0.009661	0.000830	0.000112	0.000023	0.000002

The classic pyramidal wavelet decomposition representation was used to display the ${\hat{\beta }}_{1,sl}$ corresponding to each wavelet coefficient, with the size of a given point representing its absolute value and the coloring scheme representing its sign (blue for negative, red for positive). Furthermore, if a ${\hat{\beta }}_{1,sl}$ has an associated ${\hat{\pi }}_{sl}$ that is not thresholded to zero (see Eq. 3), we highlight the region corresponding to the wavelet coefficient using the color-coding scheme in Fig. 5. We provide the genomic position of the significant locus in the “Appendix”.

### Grain yield

Grain yield in maize is a complex trait and there is a growing literature aimed at elucidating its etiology [25, 28]. The genetic underpinnings of grain yield is still not completely understood due to the small datasets available for a well-powered genome-wide analysis. Here, we used genome-wide genotypic data from 256 maizes from a large European plant consortium [25] to examine if we could replicate the lead SNPs discovered in that dataset. The lead SNPs are located on chromosome 6. Using the same exclusion criteria for SNPs and individuals as in the original article by Millet and co-workers [25], we analyzed 36,624 SNPs scattered across the 10 maize chromosomes. The data are available in the ***R*** package *statgenGWAS*.

#### Definition of regions and choice of resolution for grain yield

As with the analysis of gestational age, we also excluded regions of poor marker density in the maize dataset. This was particularly important because the data provided by Millet and colleagues [25] have a lower SNP density than those in the HARVEST dataset. We propose studying regions of 1 Mb in size, with a maximum distance of 50 kb between any two adjacent SNPs. By applying these additional criteria, we excluded $8\%$ of the SNPs and defined 246 regions across the 10 chromosomes. Moreover, as explained in the previous section, the precision of the wavelet coefficient depends on the number of non-interpolated points in a given region. It is therefore important to select a depth of analysis corresponding to approximately 10 SNPs per wavelet coefficient. Following this criterion, the median spacing between any given pair of SNPs was 3525 bp in the maize dataset. By dividing each locus of 1 Mb into $2^5 =32$ sub-regions, we have on average $\frac{10^6}{2^5}\times \frac{1}{3525} \approx 8.9$ SNPs per sub-region.

#### Model and results

We applied WS to the grain yield dataset and simulated $L_h$ and $min(p_h,p_v)$ $10^5$ times under the null using an empirical correlation matrix. Using these simulations and the steps described in the “Calibration of the hyperparameter and statistical significance” section, we obtained $\lambda ^* = 105$. We then fit a normal distribution on $L_h +\lambda ^*min(p_h,p_v)$. This distribution was then used to compute the p-values for each locus. To assess statistical significance, we used the same genome-wide significance threshold (i.e., $10^{-6}$) as in the Millet *et al. *study [25].

WS identified 44 significantly associated loci. One of these loci corresponds to the genome-wide significant locus on chromosome 6 reported by Millet and colleagues [25]. We provide in the “Appendix” the positions and p-values of the 46 genome-wide significant loci. By comparison, we did not find any of the loci reported by Boyles and colleagues [28]. It should be noted, however, that the study by Boyles and colleagues [28] did not identify any locus with a p-value below the genome-wide significance threshold.

## Discussion

This paper introduces WS as a novel and powerful alternative to the classic GWAS methodology. It offers a more flexible modeling scheme than the standard single-SNP association approach and enhances the discovery rate in case of moderate local polygenicity. We acknowledge the empirical nature of this article, as most of the simulations indicate that WS is slightly over-conservative, which might be due to our calibration criterion for $\lambda ^*$ or the shrinkage of the posterior probability. Furthermore, we acknowledge the potential limitation of the coding scheme for assigning a SNP allele as either risk-conferring or protective. The minor (less frequent) allele is conventionally coded as the risk allele, while the major allele is treated as the reference (non-risk) allele. When handling a large number of SNPs simultaneously, there is no definitive protocol for coding the alleles, especially when there is no prior knowledge of their true effects based on the results of targeted functional analyses. When the risk allele is coded wrongly, the direction of the effect of the allele may be treated as random. Under such a setting, WS would provide less power but would still be a better alternative to the single-SNP modeling. Moreover, this limitation in allele coding is not restricted to WS but is also present in all genotype-based regional tests that are not variance-based tests [19].

In future developments of WS, we plan to add new functionalities to enable GWAMAs based on the use of summary statistics from different participating cohorts, akin to what is routinely performed using the popular METAL software [29]. If each cohort defines the same genomic regions to be analyzed, as is usually done in a rare-variant meta-analysis [19]), a meta-analysis should be straightforward to perform. One can meta-analyze each region sequentially by combining the p-values for each region using Fisher’s method (see Eq. 10).

10 $$\begin{aligned} \chi ^2_{2S} = -2\sum _{s=1}^S ln(p_s) \end{aligned}$$

where *S* is the number of cohorts and $p_s$ is the p-value of the cohort *s*. However, it may be more appropriate to do a meta-analysis at the level of coefficients and then compute a new test statistic for the meta-analysis.

In addition, we aim to adapt our method to include phenotypes on non-ordered scales, e.g., blood types or psychiatric phenotypes. These phenotypes are usually analyzed in a case-control fashion and not by multinomial regression due to computational and power issues. By exploiting reverse regression, we can include such phenotypes in the predictor matrix by coding them in a similar way as in ANOVA. The modeling of the two hypotheses can be done using multivariate Gaussian, with one dimension per coefficient, instead of using simple univariate Gaussian. Furthermore, by exploiting reverse regression, we can also easily adapt this method to the multiple-phenotype GWAS setting known as phenome-wide association studies or PheWAS [30]. However, using reverse regression can reduce power, especially when analyzing phenotypes associated with large measurement errors, such as gestational duration. This can be regarded as a measurement error problem, which would result in a shrinkage of the estimated coefficients (see [31], Chapter 1, Section 1).

## Conclusions

The complexity of the test statistics makes it difficult to infer directly how power would be influenced by different parameters, such as the distance between SNPs and their LD structure. Future work should focus on exploring power behavior under different settings, including varying sample size, different percentage of variance explained, and unequally distributed effects between SNPs. The wavelet-based methodology presented here is both powerful and versatile, and lends itself easily to the analysis of other ‘omics’ data types. In future developments, we will investigate the feasibility of extending WS by adding one more level of hierarchy to enable multi-omics data analyses.

## Software

WS is distributed as an ***R*** package. In addition to the code, the package contains a data visualization tool to scrutinize any associations detected by WS. The ***R*** package is available at https://github.com/william-denault/WaveletScreening. We also provide a detailed example of a typical WS run by using simulated data. Additional details are provided in the help function *wavelet_screening*. Further, we show how to simulate $L_h$ and $min(p_h,p_v)$ under the null and how to compute $\lambda ^*$ from $L_h$ and $min(p_h,p_v)$. Finally, the user can apply the *plot_WS* function to visualize the output of *wavelet_screening*, as exemplified in Fig. 5.

## Data Availability

The GWAS dataset on gestational age from HARVEST that was used in the simulations is publicly available. They can be accessed by sending an application to the Norwegian Regional Committee for Medical Research Ethics (REK, https://rekportalen.no/), and upon approval, data administrators at MoBa (https://www.fhi.no/en/studies/moba/) need to be contacted before secured access to the data can be granted to successful applicants. The maize GWAS dataset from the Millet et al. study [25] is available in the *R* package *statgenGWAS* (https://biometris.github.io/statgenGWAS/).

## Appendix

*S1 Fig.* Covariance matrix for simulations of the $L_h$ and $min(p_h,_v)$ statistics. Left panel: covariance matrix computed using white noise (proxy covariance). Right panel: empirical covariance matrix computed using the result from GWAS. Color scale: yellow=high, red=close to 0.

## Abbreviations

SNP: Single-nucleotide polymorphism
WS: Wavelet screening
SKAT: SNP-set (sequence) kernel association test
FDWT: Fast discrete wavelet transform
LD: Linkage disequilibrium
bp: Base pair
kb: Kilobase
Mb: Megabase
GR: Genetic region
MD: Mono-direction
RD: Random direction
GWAS: Genome-wide association study
